# Multi-level characteristics of TiO_x_ transparent non-volatile resistive switching device by embedding SiO_2_ nanoparticles

**DOI:** 10.1038/s41598-021-89315-z

**Published:** 2021-05-10

**Authors:** Sera Kwon, Min-Jung Kim, Kwun-Bum Chung

**Affiliations:** Division of Physics and Semiconductor Science, Dongguk University, Seoul, 04620 Republic of Korea

**Keywords:** Electronic properties and materials, Surfaces, interfaces and thin films

## Abstract

TiO_x_-based resistive switching devices have recently attracted attention as a promising candidate for next-generation non-volatile memory devices. A number of studies have attempted to increase the structural density of resistive switching devices. The fabrication of a multi-level switching device is a feasible method for increasing the density of the memory cell. Herein, we attempt to obtain a non-volatile multi-level switching memory device that is highly transparent by embedding SiO_2_ nanoparticles (NPs) into the TiO_x_ matrix (TiO_x_@SiO_2_ NPs). The fully transparent resistive switching device is fabricated with an ITO/TiO_x_@SiO_2_ NPs/ITO structure on glass substrate, and it shows transmittance over 95% in the visible range. The TiO_x_@SiO_2_ NPs device shows outstanding switching characteristics, such as a high on/off ratio, long retention time, good endurance, and distinguishable multi-level switching. To understand multi-level switching characteristics by adjusting the set voltages, we analyze the switching mechanism in each resistive state. This method represents a promising approach for high-performance non-volatile multi-level memory applications.

## Introduction

The resistive switching device has attracted attention as a promising candidate for next-generation non-volatile memory devices, as it is can be used to achieves low power consumption, good storage capability, high-speed operation, and stable switching properties^1–4^. Many kinds of inorganic transitional metal oxide materials have been proposed for the active layer, including TiO_2_, Ta_2_O_5_, HfO_2_, and NiO^5–8^. Among them, TiO_2_ has been widely studied due to its fast switching speed, promising performance, intrinsic variety of crystal phases, and the associated richness of its switching dynamics, as well as change of the operating characteristics according to the ambient condition^9–12^. Recently, scaling down the device size and increasing the structural density of the memory array have become highly sought after targets in resistive random access memory (ReRAM) devices. The cross-bar array architecture is a widely accepted method with structural benefits, which is sandwiched by inserting active layer between the parallel bottom electrode (BE) and perpendicular to the top electrode (TE)^13–15^. Another aspect that has received considerable attention is the fabrication of a multi-level switching device, which can store multi-bit data in one cell and can enhance the physical density of the memory device^4,16,17^. In general, a resistive switching device changes electrically between a high resistive state (HRS) and a low resistive state (LRS) through the application of electrical inputs. By contrast, the multi-level switching device has various HRS or LRS; specifically, the multi-level switching characteristics can be generated by either modifying the length of the gap between the conductive filament (CF) and electrode, which is controlled by adjusting the stop voltage during the RESET process, or by controlling the diameter/density of the CF during the LRS process^18–20^. Recent research has indicated that the switching performance of a switching device can be improved by incorporating nanostructures into the matrix of the switching layer. Tsigkourakos et al. has reported the fabrication of a TiO_2-x_-based resistive switching device by embedding Pt nanocrystals, and this device achieved low power operation current and obtained multi-level switching characteristics by controlling the compliance current^21^. Yoon et al. embedded Ru nanodots into TiO_x_ thin film to achieve resistive switching uniformity among TiO_2_ memory cells^22^. The embedment of metal nanostructures has attracted substantial attention, as it improves the resistive switching performance by controlling the size and shape. However, metal nanostructure-embedded switching device has a crucial issue for fabrication of transparent switching devices, due to the inherent opaqueness of metal; this means it is unfeasible to use such devices in next-generation memory devices. Transparent memory devices are extremely useful due to their potential applications in the field of transparent electronics. Herein, we attempt to achieve fully transparent non-volatile memory devices, as well as multi-level switching characteristics, by using SiO_2_ nanoparticles (NPs).

**Figure 1:**
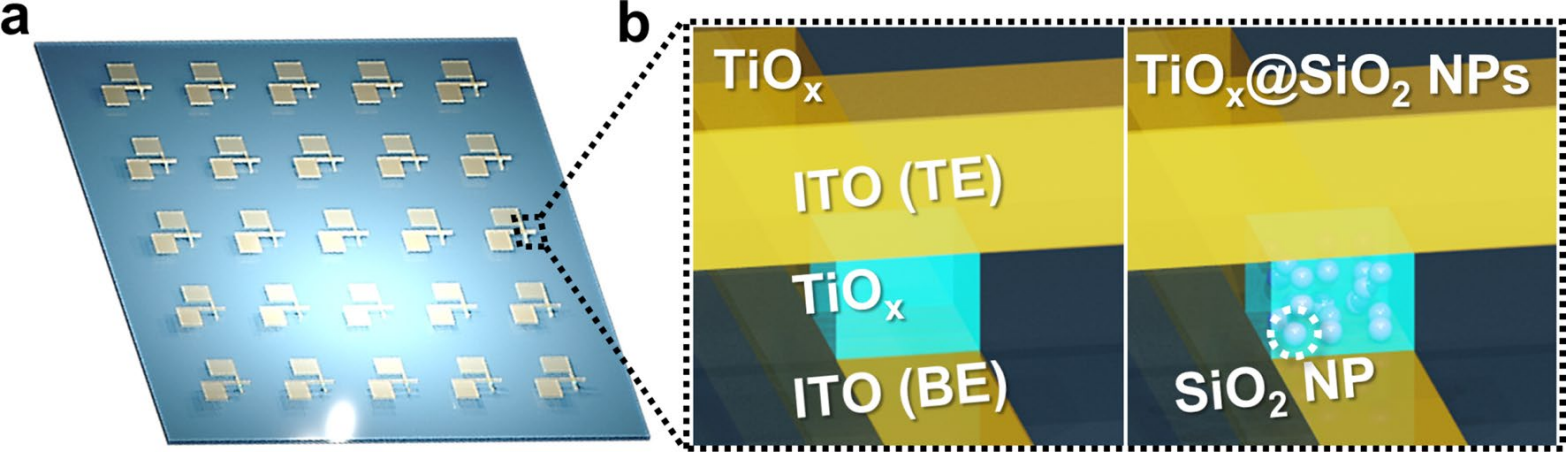
(**a**) Schematic illustration of cross-bar arrays of the TiO_x_ switching device. (**b**) Enlarged view of the cross-section region of the switching device unit cell.

In this study, we prepared a multi-level switching device with transparent characteristics by embedding SiO_2_ NPs into the TiO_x_ matrix (structure with ITO/SiO_2_ NPs-embedded TiO_x_/ITO) on glass substrate using a solution process, as shown in Fig. 1. The SiO_2_ NPs-embedded TiO_x_ device showed high transmittance as well as outstanding electrical performance, including a large on/off current ratio, long retention time, good endurance, and multi-switching characteristics.

## Results and discussion

**Figure 2:**
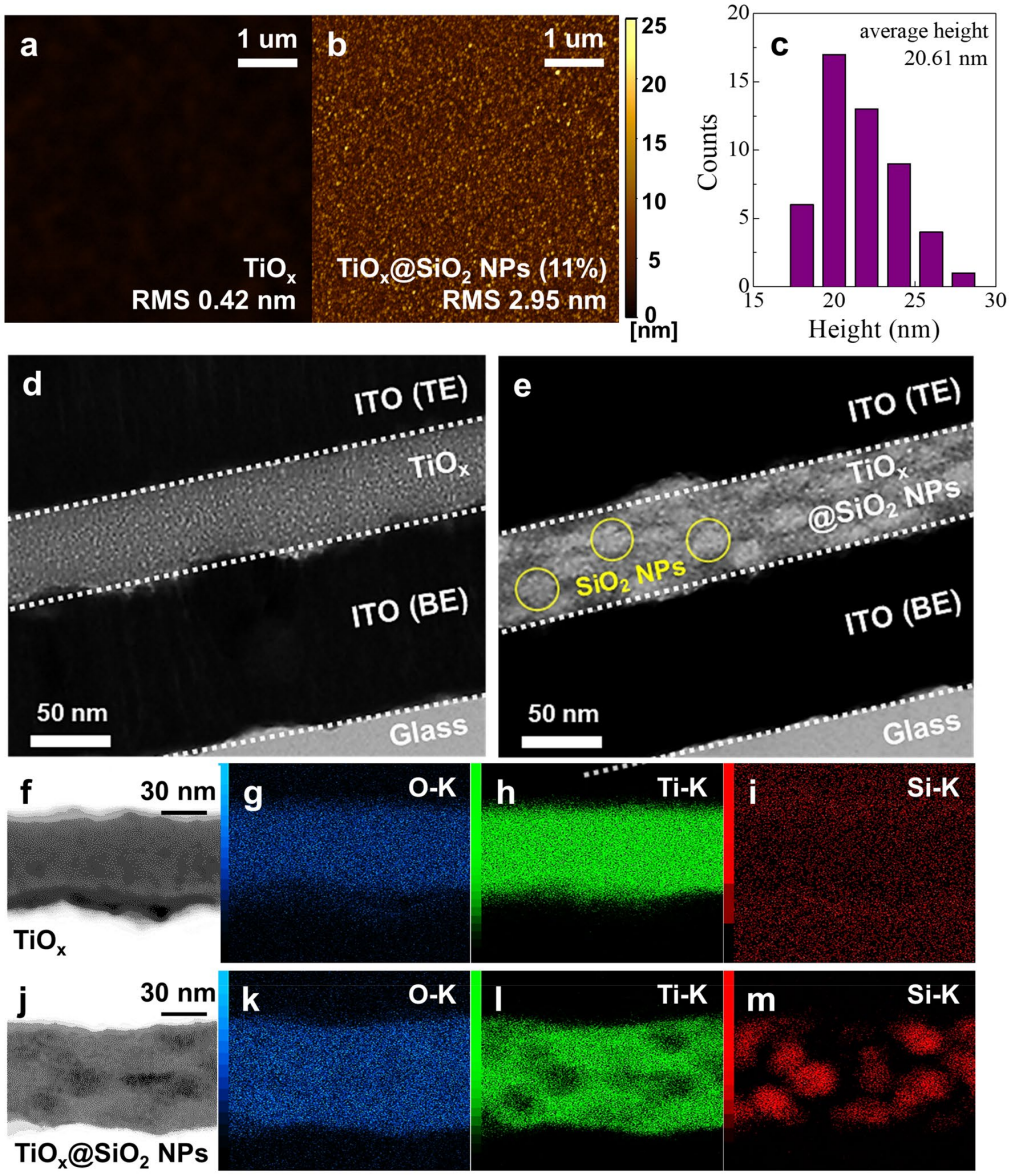
(**a**,**b**) AFM topographic images, (**c**) average height of SiO_2_ NPs, (**d**,**e**) cross-sectional TEM images, and (**f**–**i**) HAADF-STEM and EDS spectra of TiO_x_ and (**j**–**m**) TiO_x_@SiO_2_ NPs structures.

Figure 2a,b show the surface morphologies of the TiO_x_ and TiO_x_@SiO_2_ NPs films, which were obtained using atomic force microscopy (AFM) measurement. It can clearly be seen that the nano-particles with a sphere shape are well embedded into the TiO_x_ matrix. In addition, the root-mean-square (RMS) roughness values for TiO_x_ and TiO_x_@SiO_2_ NPs are changed from 0.42 to 2.95 nm, respectively. Further, the average height of SiO_2_ NPs is approximately 20.61 nm, as shown in Fig. 2c. Moreover, TEM measurement was conducted to examine the cross-sectional structures of the TiO_x_ and TiO_x_@SiO_2_ NPs switching devices. As shown in Fig. 2d,e, the TEM image of the TiO_x_ device clearly shows stacking of ITO/TiO_x_/ITO. On the other hand, the brighter areas with a spherical shape that are well distributed in the region of the TiO_x_@SiO_2_ NPs layer are estimated to be SiO_2_ NPs. For a detailed analysis of the bright areas in the TiO_x_@SiO_2_ NPs layer, high-angle annular dark-field scanning TEM (HAADF-STEM) was taken across the TiO_x_ and TiO_x_@SiO_2_ NPs, as shown in Fig. 2f,j. According to HAADF-STEM, the heavy element shows bright contrast, while the light element shows dark contrast^23^. In the HAADF-STEM image of TiOx@SiO_2_ NPs, the darker region that exists in the shape of a sphere in the TiO_x_ matrix corresponds to SiO_2_ NPs. To investigate the chemical species, EDS spectra are taken. In Fig. 2g–i, only Ti and O elements are detected in the case of the TiOx device, whereas the TiO_x_@SiO_2_ NPs device is composed of Ti, O, and Si as shown in Fig. 2k–m. Based on the cross-sectional information, the distribution of SiO_2_ NPs in TiO_x_ is definitively confirmed.

**Figure 3:**
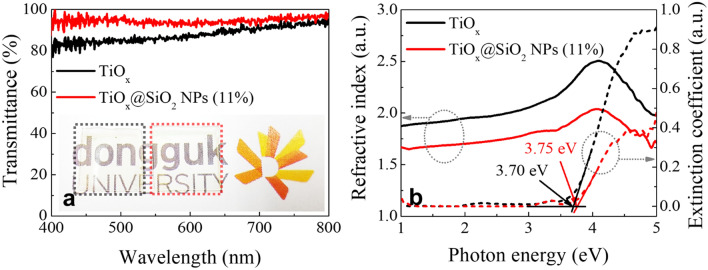
(**a**) Optical transmittance and (**b**) optical constants of the TiO_x_ and TiO_x_@SiO_2_ NPs films. Inset shows the high transparency of the TiO_x_ (left) and TiO_x_@SiO_2_ NPs (right) switching devices.

Figure 3a shows the transmittances of the ITO/TiO_x_/ITO device and the ITO/TiO_x_@SiO_2_ NPs/ITO structures. As shown in the inset in the Fig. 3a, the two devices are both highly transparent. To compare the optical transmittances values of the TiO_x_ and TiO_x_@SiO_2_ NPs devices, we measured the optical transmittance in the visible range from 400 to 800 nm, and we ultimately obtained respective values of 87 and 95%. It is concluded that the optical transmittance is improved by embedding SiO_2_ NPs into TiO_x_. Figure 3b shows the refractive index and extinction coefficient of TiO_x_ and TiO_x_@SiO_2_ NPs, which were obtained using a four-phase model comprising glass substrate, ITO layer, TiO_x_ layer, and ambient layer^24^. For the TiO_x_ film, the refractive index was 1.96, which is smaller than that of conventional TiO_2_ film (which ranges from 2.4 to 2.75 at 550 nm)^25^. As research has shown that the refractive index can be related to the film density, we consider that a decrease in the refractive index for the prepared TiO_x_ film implies a lower density than that of conventional TiO_2_ film^26,27^. In the TiO_x_@SiO_2_ NPs, the value of the refractive index decreases to 1.72, as it is affected by the embedment of the small refractive index of SiO_2_ NPs (~ 1.47)^28^. Embedding SiO_2_ NPs into the TiO_x_ matrix also leads to a decrease in the extinction coefficient, which represents a decrease in absorption. The optical band gap can be extracted by extrapolating the linear portion of the extinction coefficient. The optical band gap of TiO_x_ and TiO_x_@SiO_2_ NPs is found to be 3.70 and 3.75 eV, respectively. These values are consistent with the TiO_x_ films made by the solution process, and the TiO_x_ and TiO_x_@SiO_2_ NPs are both completely transparent in the visible region^29^.

**Figure 4:**
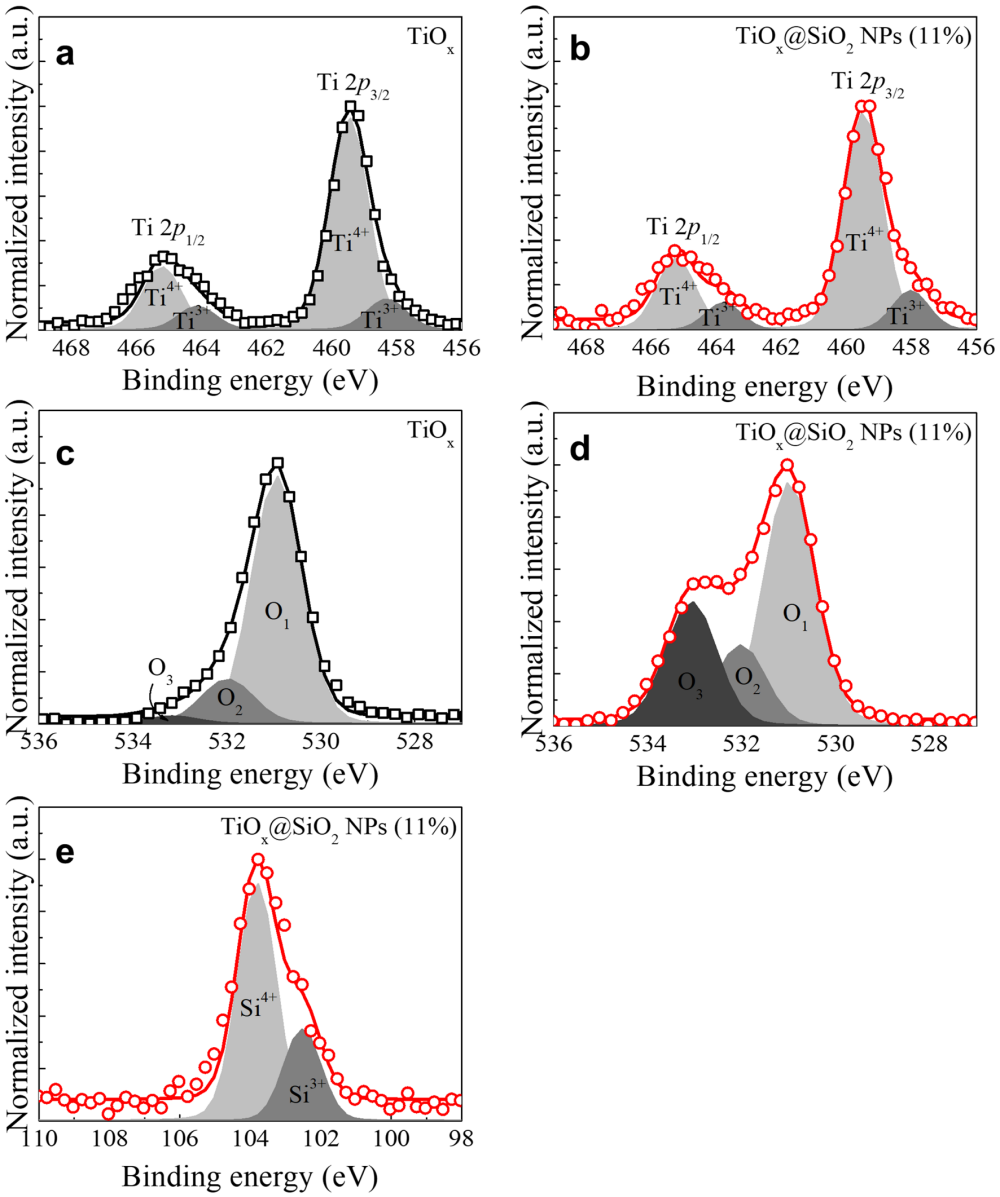
XPS core-level spectra of (**a**,**b**) Ti 2*p*, (**c**) and (**d**) O 1*s* for TiO_x_ and TiO_x_@SiO_2_ NPs films, and (**e**) Si 2*p* for TiO_x_@SiO_2_ NPs film.

Figure 4a–e respectively show the Ti 2*p*, O 1*s*, and Si 2*p* core-level spectra of the TiO_x_ and TiO_x_@SiO_2_ NPs films. The XPS analysis was conducted after surface sputtering for 10 s to eliminate the carbon contamination, which was performed by Ar sputtering at 500 W. The survey spectra were measured to acquire accurate compositions of the TiO_x_ and TiO_x_@SiO_2_ NPs films (Fig. S1). In the case of TiO_x_@SiO_2_ NPs, the TiO_x_ matrix consists of 11.5% Si. To elucidate the chemical bonding states, the core-level spectra of Ti *2p*, O 1*s*, and Si 2*p* were normalized, then deconvoluted into Gaussian peaks. Figure 4a,c show the Ti 2*p* spectra, wherein the two peaks centered at 459.15 and 453.45 eV can be attributed to Ti 2*p*_1/2_ and Ti 2*p*_3/2_, respectively. The separation between these two peaks is calculated to be ~ 5.7 eV, which suggests the existence of Ti^4+^ oxidation states in the TiO_2_^30^. The shoulder peaks at 458.15 and 452.45 eV can be correlated with the Ti^3+^ oxygen deficient states in TiO_x_, which originate from the reduction of Ti^4+^ by free electrons donated by oxygen vacancies^29,31^. In the TiO_x_@SiO_2_ NPs, the intensity of the Ti^3+^ peaks slightly increases relative to those of the TiO_x_, which is attributed to the TiO_x_@SiO_2_ NPs sample having more oxygen deficient state in the TiO_x_ matrix. The O 1*s* peaks are composed of three Gaussian peaks corresponding to the Ti–O bonds at 531 ± 0.2 eV (O1), oxygen deficient states at 532 ± 0.2 eV (O2), and hydroxyl groups at 533 ± 0.2 eV (O3), respectively, as shown in Fig. 4b,d^32^. Upon embedding the SiO_2_ NPs into the TiO_x_ matrix, the O2 peak of the TiO_x_@SiO_2_ NPs drastically increases compared to those of the TiO_x_. There is also an increase in the O3 peak, which is associated with an increased number of residual surface hydroxyl groups; however, this is disputable, because the binding energies of the Si–O bonds overlap the hydroxyl groups attributed to O3^33^. In our explanation, the effect of the surface hydroxyl groups is negligible, because the dominating mechanism for the TiO_x_-based switching device comes from the oxygen vacancies. Si 2*p* is composed of regular SiO_2_ (Si^4+^) as well as a substantial amount of oxygen deficient states in SiO_x_ (Si^3+^), as shown in Fig. 4e^34^. This indicates that the TiO_x_@SiO_2_ NPs has a higher composition of oxygen vacancies than TiO_x_. These results can be attributed to the migration of oxygen ions from the TiO_x_ matrix into SiO_2_ NPs, which is caused by the higher bond dissociation energy of Si–O (799.6 ± 13.4 kJ/mol) than that of Ti–O (666.5 ± 5.6 kJ/mol); therefore, the embedment of SiO_2_ NPs is shown to produce the oxygen deficient states in the TiO_x_ matrix^35^. In the TiO_x_-based ReRAM, defects such as oxygen deficient states play an important role in the switching characteristics, which is strongly related to the migration of oxygen vacancies and the formation/rupture of the conductive path caused by the application of an external electric field^36^. It is also known that the physical size of the conductive filament depends on the concentration of oxygen vacancies. Thus, the size of the conductive filament for the TiO_x_@SiO_2_ NPs is expected to be wider than that of the TiO_x_ device, and various electrical characteristics, such as multi-level switching, can be expected.

### Electrical characteristics

**Figure 5:**
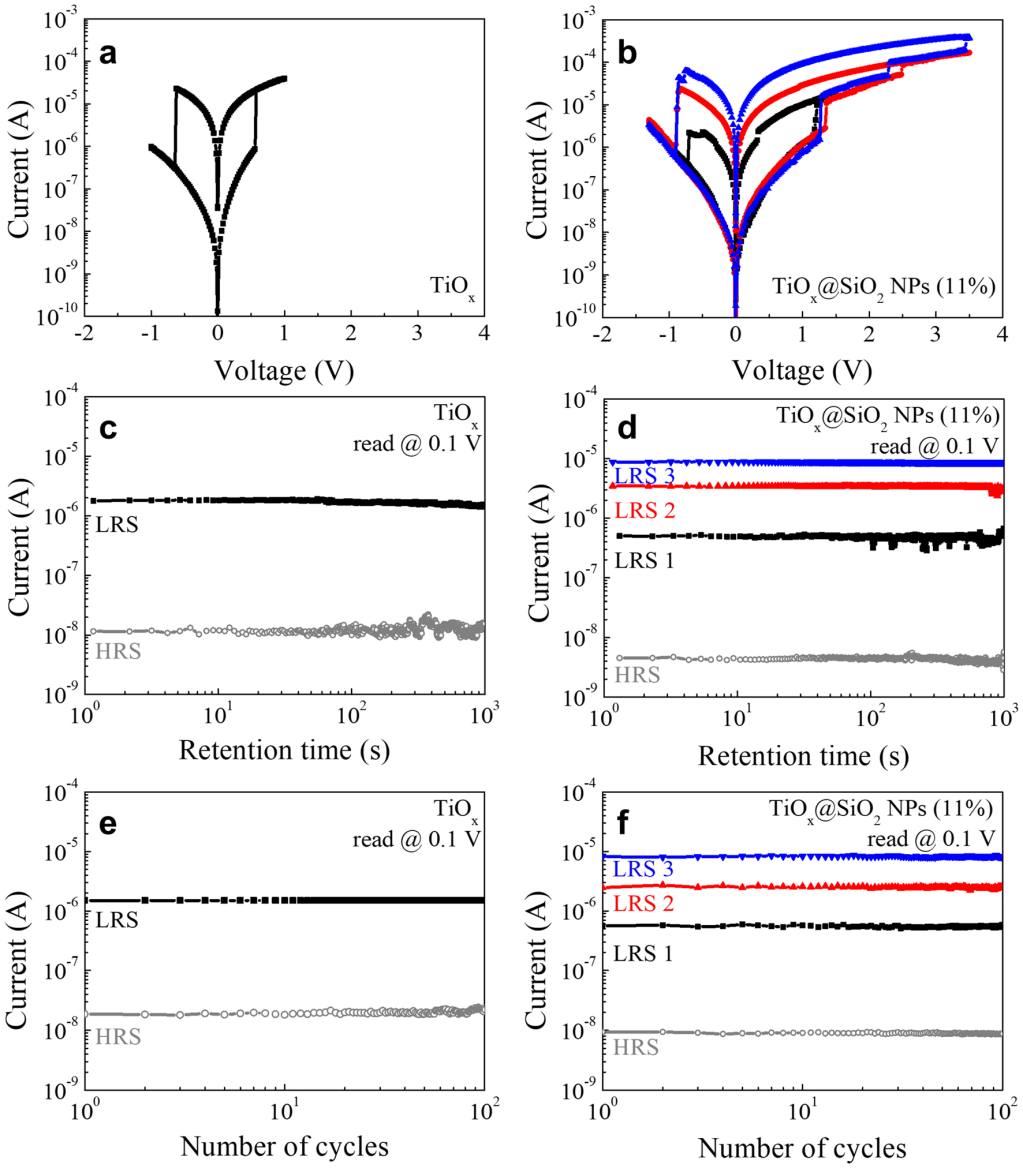
(**a,b**) Resistive switching curves, (**c,d**) retention performance, and (**e,f**) endurance performance of the TiO_x_ and TiO_x_@SiO_2_ NPs switching devise.

Figure 5a,b show the typical I–V curves under voltage sweeping for the TiO_x_ and TiO_x_@SiO_2_ NPs switching devices after the electro-forming process (Fig. S2). Initially, the TiO_x_-based switching device is in HRS, because of the Schottky barrier at the interface of the electrode/TiO_x_. In our device structure with ITO/TiO_x_/ITO, the electron transport is limited due to the difference between the work-function of the ITO electrode (~ 4.4 eV) and TiO_x_ (~ 3.8 eV)^37,38^. As shown in Fig. 5a,b, both devices exhibit bipolar resistive switching characteristics. Regarding the TiO_x_ device, it switches from HRS to LRS, and the SET process is achieved by sweeping the voltage from 0 to 0.56 V. When sweeping the voltage from 0 to − 0.62 V, the state changes from LRS to HRS, and the RESET process can be obtained. It is known that TiO_2_ has the coexistence of bipolar resistive switching characteristics, thus they can be achieved SET/RESET process in both polarity regardless of the voltage polarity of the electro-forming process^39^. For the TiO_x_@SiO_2_ NPs device, it is clearly indicated that the multi-step SET process can be obtained by controlling the voltage. When the positive voltage sweeps from 0 to 1.2 V, the RS transits from the HRS to the lowest LRS, denoted as LRS1. Upon increasing the voltage to 2.2 V, the intermediate LRS (LRS2) can be achieved. And then, further increasing the voltage to 3.3 V, the highest LRS can be obtained, it indexed to LRS3. From sweeping the positive voltage, we obtain multi-level switching characteristics with four storage levels. During the negative voltage sweeps, the RS changes from LRS to HRS. It can be attributed that the SiO_2_ NPs are a little more existed at the interfaces of TiO_x_@SiO_2_ NPs/top electrode than that of interfaces of TiO_x_@SiO_2_ NPs/bottom electrode (Fig. S3). When the electrons are injected from top electrode into TiO_x_@SiO_2_ NPs layer, SiO_2_ NPs suppress the carrier transport, thus, the switching device maintains the HRS under negative voltage. Consequentially, SiO_2_ NPs with high bandgap provides the asymmetric potential barrier for the bipolar resistive switching^40^. Another interesting result is that the current level slightly decreases compared to that of the TiO_x_ device, meaning that the ratio of HRS/LRS is also improved. This result indicates that the SiO_2_ NPs act as an insulating layer. To ensure that the multi-level switching characteristics are due to the embedment of SiO_2_ NPs, the switching mechanism was examined as a function of the composition of SiO_2_ NPs, and it was found that the multi-level switching characteristics appeared slightly at the low composition (Fig. S4). Based on the I–V analysis, it can be concluded that the embedment of SiO_2_ NPs into the TiO_x_ matrix can significantly improve the resistive switching performance, such as multi-level switching, and obtain a high ratio of HRS/LRS for TiO_x_-based switching devices.

To examine the stability of the device, as shown in Fig. 5c,d, the retention was studied by probing the RS changes in the HRS and LRS states for 10^3^ s at room temperature, and measurements were performed by separately reading the current at 0.1 V after the SET and RESET processes. The TiO_x_ device maintained a stable HRS/LRS ratio over 10^2^ for 10^3^ s. The TiO_x_@SiO_2_ NPs device exhibits four well-defined RSs (HRS, LRS 1, LRS 2, and LRS 3), each of which is observed for 10^3^ s. Further, to examine the endurance performance, the RS cycling test was conducted for 10^2^ cycles after the SET and RESET processes, as shown in Fig. 5e,f. The two devices both show stable HRS/LRS ratios for 10^2^ cycles. Specifically, in the case of the TiO_x_@SiO_2_ NPs, multiple RSs are well defined for 10^2^ cycles. The long retention time and high endurance indicate the high reliability and non-volatile nature of the TiO_x_-based resistive switching devices.

### Switching mechanism

**Figure 6:**
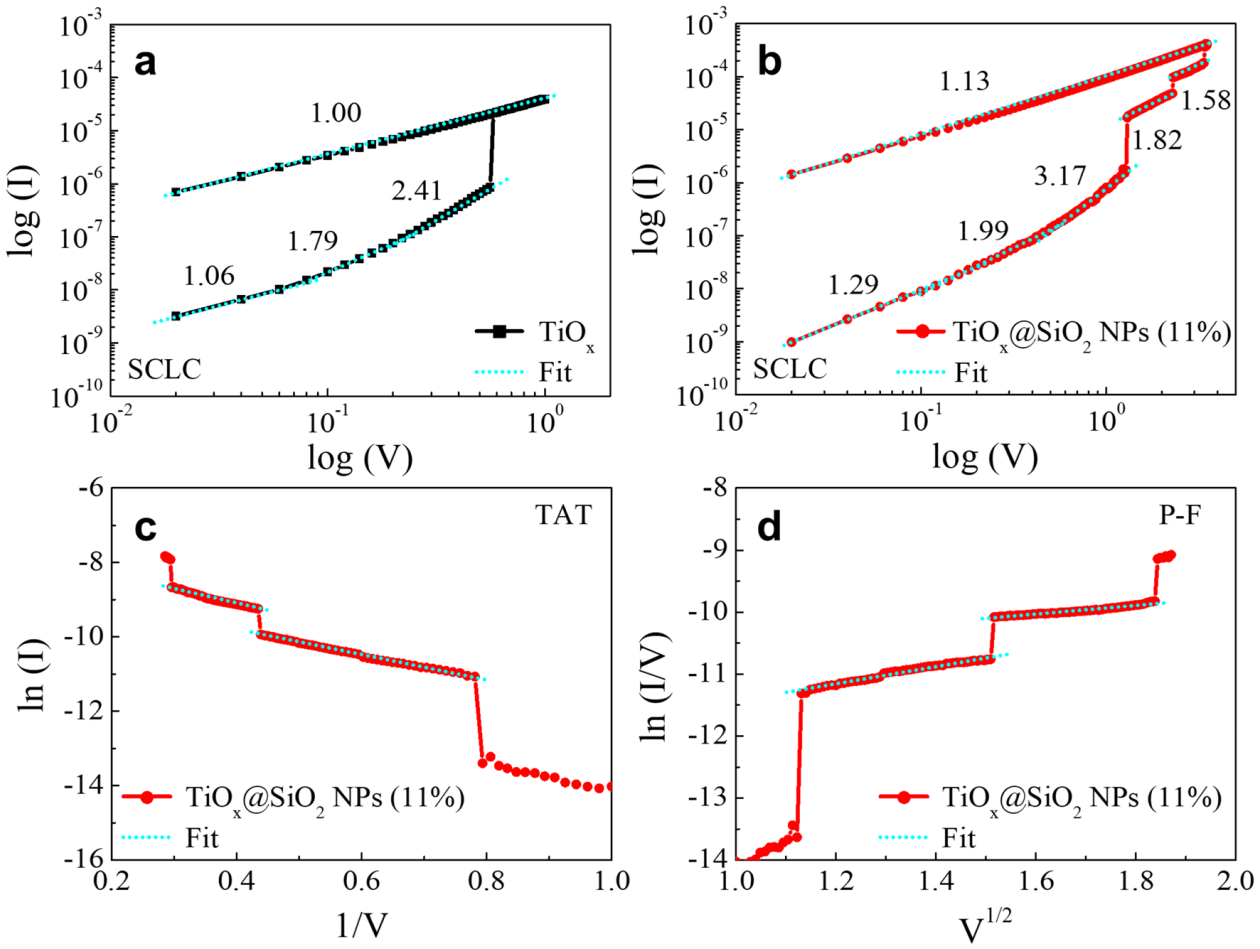
Log I–log V plot of I–V curves of the (**a**) TiO_x_ and (**b**) the TiO_x_@SiO_2_ NPs switching devices. The TiO_x_@SiO_2_ NPs implies (**c**) trap-assisted tunneling, and (**d**) Poole–Frenkel emission in the high-voltage region.

The conduction mechanism of the TiO_x_ and TiO_x_@SiO_2_ NPs devices can be obtained by analysing the I–V curves. For the TiO_x_ device, the fitting results for HRS suggest that the charge transport mechanism is in good agreement with a trap-controlled space charge limited conduction (SCLC), as shown in Fig. 6a. The I–V curve consists of three regions with slopes of (1.06, 1.79, and 2.41), which respectively relate to the Ohmic region (I ∝ V) dominated by thermally generated free carriers; the Child’s law region (I ∝ V^2^), in which traps are filled by electrons; and the steep increase region (V ∝ I^n^, n > 2)^29,41–43^. In the TiO_x_-based switching device, oxygen vacancies in the TiO_x_ matrix serve as electron traps, and they lead to the formation or rupture of the conductive path. Therefore, the migration of oxygen vacancies is an important role in the deviation of slopes. In the high-voltage region, all traps in the TiO_x_ matrix are filled by electrons, then electrons flow through the conduction band of TiO_x_, after which the TiO_x_ device achieves the SET process. For the RESET process, the slope is closer to ~ 1.00, which is attributed to the Ohmic behavior being the dominant carrier transport mechanism during the RESET process. Both the Ohmic and SCLC mechanisms are associated with the bulk controlled mechanism, meaning that the TiO_x_ device is properly explained by the conductive filament model. In the case of the TiO_x_@SiO_2_ NPs device, it is important to understand the conduction mechanism of each RS. Figure 6b shows the log I – log V plotting result of the TiO_x_@SiO_2_ NPs device, and the carrier transport mechanism of LRS 1 also corresponds to a trap-controlled SCLC in the range from 0 to 1.28 V^44^. Upon further increasing the voltage above 1.28 V, the trap-controlled SCLC is not explained, due to the decreases in the fitted lines to 1.82 and 1.58. To validate the transport characteristics of the LRS 2 and LRS 3, we apply the trap-assisted tunnelling (TAT) model and Poole–Frenkel (P–F) emission, as shown in Fig. 6c,d, respectively. These conduction mechanisms are typically calculated when examining oxide materials with a high concentration of traps. The I–V curve is linearly fitted by the TAT model, which describes a plot of ln (I) − 1/V, as shown in Fig. 6c. The TAT is attributed to the traps, such as oxygen vacancies, which help the electrons tunnel from cathode to anode^45^. In the high-voltage region, the discrete trap sites are created in the switching stack, and carrier conduction becomes possible through the trap-to-trap tunnelling process. Moreover, the P–F emission with a plot of ln (I/V) − V^1/2^ displays a linear relationship in the high-voltage region, as shown in Fig. 6d. The P–F emission results indicate that the carriers trapped in trap sites obtain enough energy, such as a high electric field or high temperature, that the trapped carriers are excited from the energy barriers of the traps to the conduction band^29^. It is known that the transition of P–F emission, which is typically at high-voltage, is much higher because of the much closer spacing of the traps. Therefore, the electron occupation probability of the nearest traps is very low, since all the electrons are quickly swept out through P–F emission^46^. In our case, multiple RSs can be attributed to various conduction mechanisms, including the TAT model and P–F emission under a high electric field. Based on these analyses, we can understand the electron conduction mechanism in each RS. The conduction mechanism for LRS can also be explained by Ohmic conduction: In the low-voltage region, lots of traps in the TiO_x_ matrix and SiO_2_ NPs connect with each other, and this contributes to Ohmic conduction. Upon increasing the voltage, the conductive path into the TiO_x_ matrix and SiO_2_ NPs begins to be formed. When large current flows through the conductive path, the conduction mechanism in LRS is explained by Ohmic behavior with a slope of 1.13, as shown in Fig. 6b.

**Figure 7:**
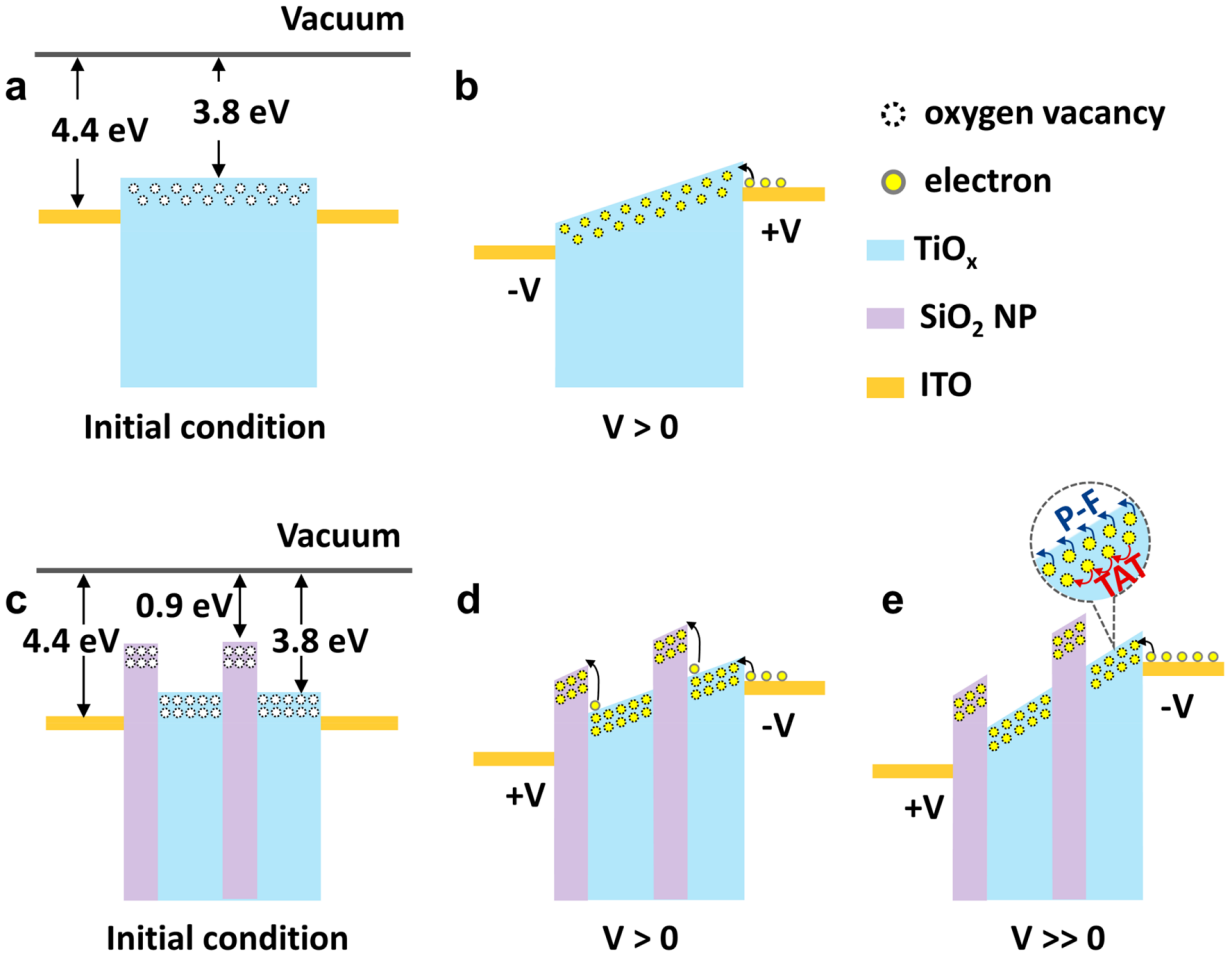
Schematic energy band diagrams for (**a,b**) the TiO_x_ device and (**c**–**e**) the TiO_x_@SiO_2_ NPs under initial states and SET process.

Figure 7a–e show schematics of the energy band diagrams of the TiO_x_ and TiO_x_@SiO_2_ NPs devices, respectively, in the initial and SET states. As shown in Fig. 7b, with the application of positive voltages to TE, the traps below the TiO_x_ conduction band are gradually filled with electrons. After all of the traps are filled (i.e., after exceeding the trap-filled limit voltage, V_TFL_), the current rapidly increases, and the TiO_x_ device switches from HRS to LRS. When negative voltage is applied to the TE, de-trapping of the oxygen vacancies occurs, and the TiO_x_ device transitions from LRS to HRS. The dominant mechanism in the TiO_x_@SiO_2_ NPs device is almost the same as that in the TiO_x_ device. In the initial state of the TiO_x_@SiO_2_ NPs device, the energy barrier exists at TiO_x_/SiO_2_ NPs, as shown in Fig. 7c. Therefore, compared to the TiO_x_ device, more sufficient voltage is needed to pass across TiO_x_ and SiO_2_ NPs. Upon applying voltages to the TiO_x_@SiO_2_ NPs device, the electrons can migrate from TiO_x_ to SiO_2_ NPs, and thus overcome the energy barrier of TiO_x_/SiO_2_ NPs. The electrons move along the tilted conduction band of the TiO_x_ layer, and then the TiO_x_ device reaches LRS (LRS 1). When the higher voltage is applied to the TiO_x_ device, the trapped electrons could either hop to other trap sites or be excited to the conduction band. As a result, the current level abruptly increases, and the TiO_x_ device eventually reaches higher current levels (LRS 2 and LRS 3). When the negative voltage is applied to the electrode, the conductive filament ruptures, and the device switches from LRS to HRS.

## Conclusion

In conclusion, we have demonstrated an improvement in the optical and electrical characteristics of a TiO_x_ resistive switching device with the embedment of SiO_2_ NPs, which are structured with ITO/TiO_x_@SiO_2_ NPs/ITO on glass substrate. The TiO_x_@SiO_2_ NPs device structure shows a high transmittance over 95% in the visible range. The embedment of SiO_2_ NPs into the TiO_x_ matrix induces higher oxygen vacancies in the TiO_x_ matrix; further, because Si–O has a higher bond dissociation energy than Ti–O, it determines the formation and rupture of the conductive path through the application of an electric field. The TiO_x_@SiO_2_ NPs device exhibits stable bipolar resistive switching characteristics, and a distinguishable multi-level of four states (three of LRS and one of HRS) can be obtained by applying voltage. Moreover, the device shows a long retention time for 10^3^ s as well as good endurance for 10^2^ cycles. The dominant switching mechanism is based on the conductive filament model, which includes the trap-controlled SCLC. After all the traps are filled out, the TAT and P–F emission gradually become dominant in the high-voltage region. The embedment of SiO_2_ NPs in the TiO_x_-based device can lead to improved electrical characteristics, such as multi-level switching, as well as low-voltage operation with stable resistive states, thus making it a promising method for future non-volatile memory devices.

## Methods

### Synthesis and fabrication

TiO_x_ was synthesized using the sol–gel method, which is based on the hydrolysis of alkoxides in alcoholic solutions in the presence of an acid catalyst. Titanium isopropoxide (TTIP, Ti[OCH(CH3)2]4, Aldrich Chemical Co., 97%), ethanol (C_2_H_5_OH, Daejung 99.9%), and nitric acid (HNO_3_, Merck, 70%) were used as the starting materials, and distilled (DI) water was used for hydrolysis. The molar ratio of the starting solution was 1 of TTIP, 1 of DI water, 25 of ethanol, and 0.2 of nitric acid^47^. The solution of nitric acid, DI water, and half ethanol was added dropwise to the solution of TTIP and ethanol at 0 °C under continuous vigorous stirring. The transparent solution finally resulted, and it was diluted using ethanol with a ratio of 1:1.

### Fabrication of switching device

To fabricate the TiO_x_-based switching memory device with a cross-bar array architecture, a glass substrate was first cleaned by wet cleaning through ultra-sonication for 10 min each in acetone, isopropyl alcohol, and DI water, before being dried in an oven at 80 °C for 20 min. Next, the promoter was coated on the glass substrate at 1000 rpm for 10 s and 5000 rpm for 60 s to enhance the adhesion of the photoresist, after which negative photoresist (5214) was coated at the same condition, followed by soft baking at 110 °C for 140 s. Then, the glass substrate was exposed to UV light for 3 s with photomask using an aligner. After being baked again at 120 °C for 200 s, the glass substrate was exposed again to UV light for 7 s without photomask, after which it was soaked in developer for 60 s. Subsequently, ITO layer was deposited on the patterned photoresist using direct current sputtering, and the substrate was soaked in acetone for lift-off of the photoresist, leading to the BE patterns. In the next step, 50 nm-thick TiO_x_ film was coated on the patterned BE of ITO by the spin coating method at 5500 rpm for 60 s. For the one-step solution process, 230 μL of SiO_2_ NPs (Nanocomposix, 20 ± 4 nm) and 100 μL of diluted TiO_x_ solution were mixed together under continuous stirring for 15 min, after which the mixed solution was coated with the same condition. Next, the coated TiO_x_ and SiO_2_ NPs-embedded TiO_x_ (TiO_x_@SiO_2_ NPs) were dried at 80 °C for 20 min in an oven. All of the TiO_x_ and TiO_x_@SiO_2_ NPs films had an amorphous structure (Fig. S5). Then, to obtain the TE pattern, the same process used for photoresist patterning and ITO deposition was performed repeatedly on the TiO_x_ and TiO_x_@SiO_2_ NPs layers. Finally, the cross-bar array ReRAM structures with an active device area of 100 μm^2^ were obtained.

### Characterization

The structural characteristics were observed using atomic force microscopy (AFM, Bruker Corp. N8 NEOS) measurement. The scanning was performed using non-contact mode and a scan size of 5 × 5 um^2^. The cross-sectional specimens were prepared using a focused ion beam (FIB, FEI Helios 650) system, then examined using field effect transmission electron microscopy (TEM, JEOL Ltd. JEM-F200) with energy-dispersive spectroscopy (EDS). The optical transmittance was measured with ultraviolet–visible (UV–Vis, TEC5 MultiSpec) spectroscopy in the range from 400 to 800 nm. The thickness and optical constant of the TiO_x_ and TiO_x_@SiO_2_ NPs layers were examined through spectroscopic ellipsometry (SE, J. A. Woollam-VASE) measurement from 1 to 5 eV with an incident angle of 65°. To accurately investigate the composition and chemical state of TiO_x_ and TiO_x_@SiO_2_ NPs, x-ray photoelectron spectroscopy (XPS, ESCA Veresprobe II) was conducted using a monochromatic Al Kα source (*hv* = 1486.7 eV) with a pass energy of 29.5 eV. The current–voltage (I–V) was measured using a semiconductor analyzer (Keithley-4200). During the I–V measurement, the bias was applied to the TE of ITO, and the BE of ITO was grounded under ambient conditions.

## Supplementary Information

Supplementary Information.
