# Supplementary material for: Multi-level characteristics of TiOx transparent non-volatile resistive switching device by embedding SiO2 nanoparticles
Source: Sci Rep. 2021 May 10;11:9883. doi: 10.1038/s41598-021-89315-z (PMC8110581; doi:10.1038/s41598-021-89315-z)
Supplement: Supplementary file 1 — Supplementary Information. [file 41598_2021_89315_MOESM1_ESM.docx]

**Multilevel characteristics of TiO_x_ transparent non-volatile resistive switching device by embedding SiO_2_ nanoparticles**

Sera Kwon^1^, Min-Jung Kim^1^, Kwun-Bum Chung^1,*^

^1^Division of Physics and Semiconductor Science, Dongguk University, Seoul, 04620, Republic of Korea

e-mail: kbchung@dongguk.edu


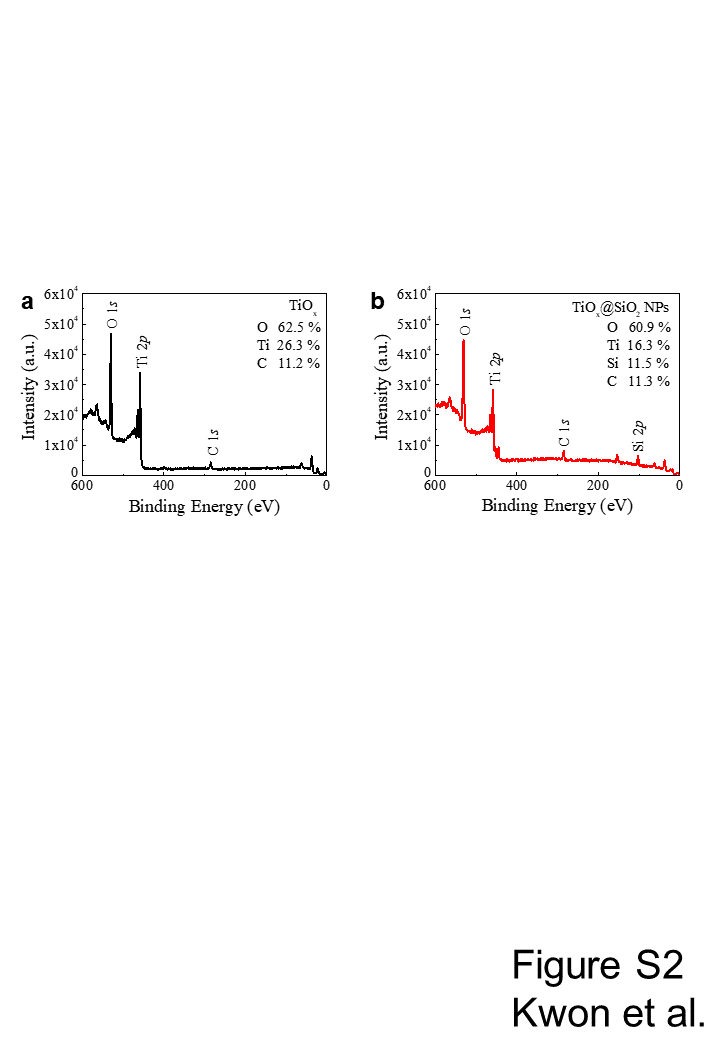


**Figure S1**. Survey spectra and compositions of (a) TiO_x_ and (b) TiO_x_@SiO_2_ NPs films.


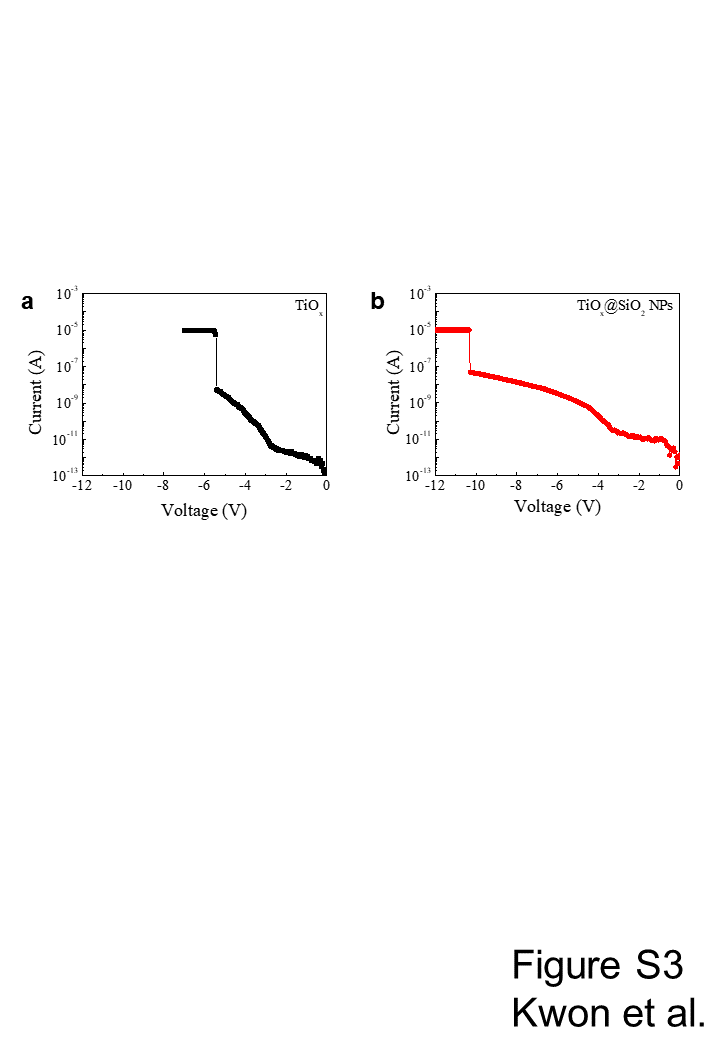


**Figure S2**. Electro-forming process of (a) TiO_x_ and (b) TiO_x_@SiO_2_ NPs switching devices.


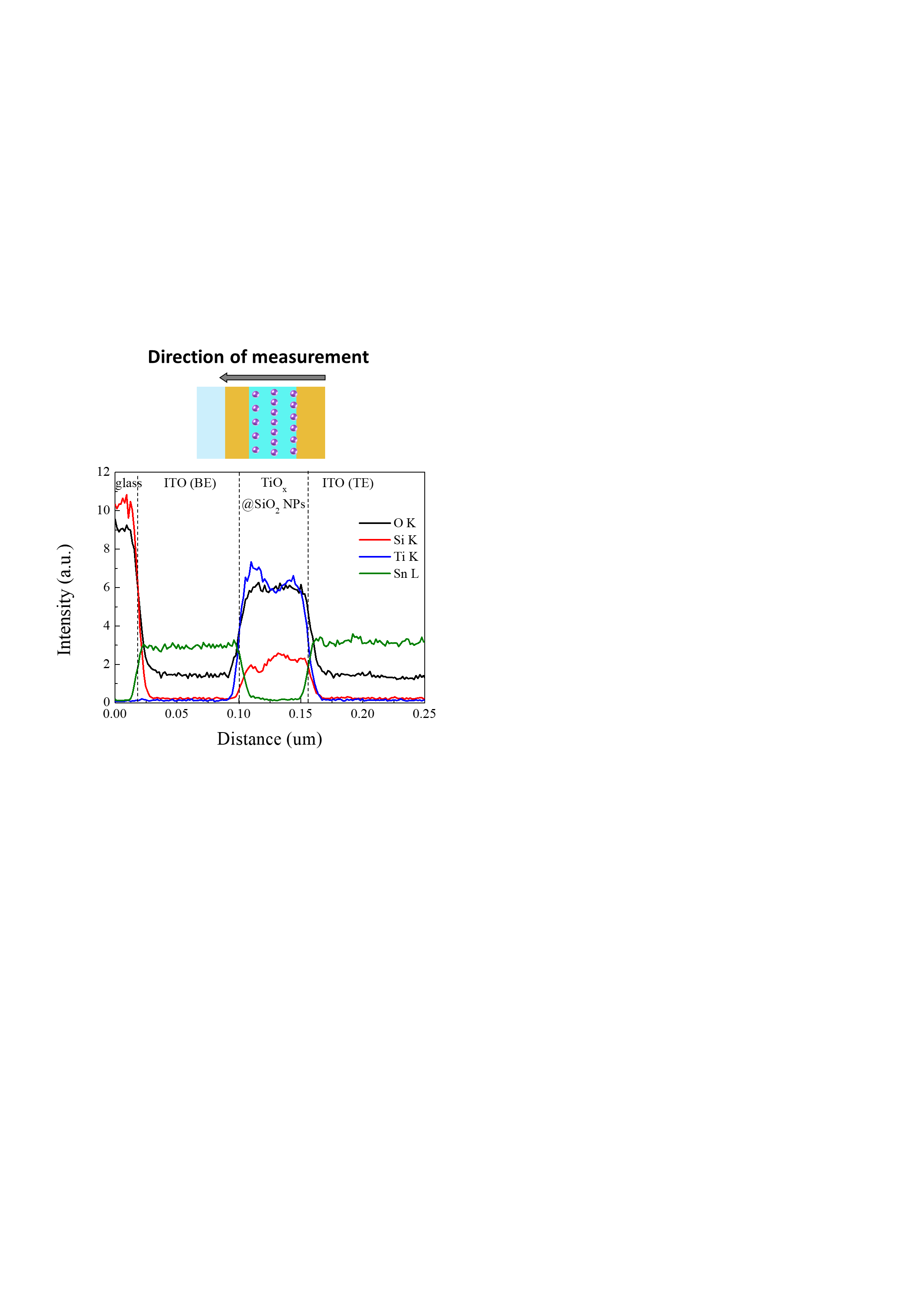


**Figure S3**. EDS analysis in the depth direction of ITO/TiO_x_@SiO_2_ NPs/ITO on glass substrate.


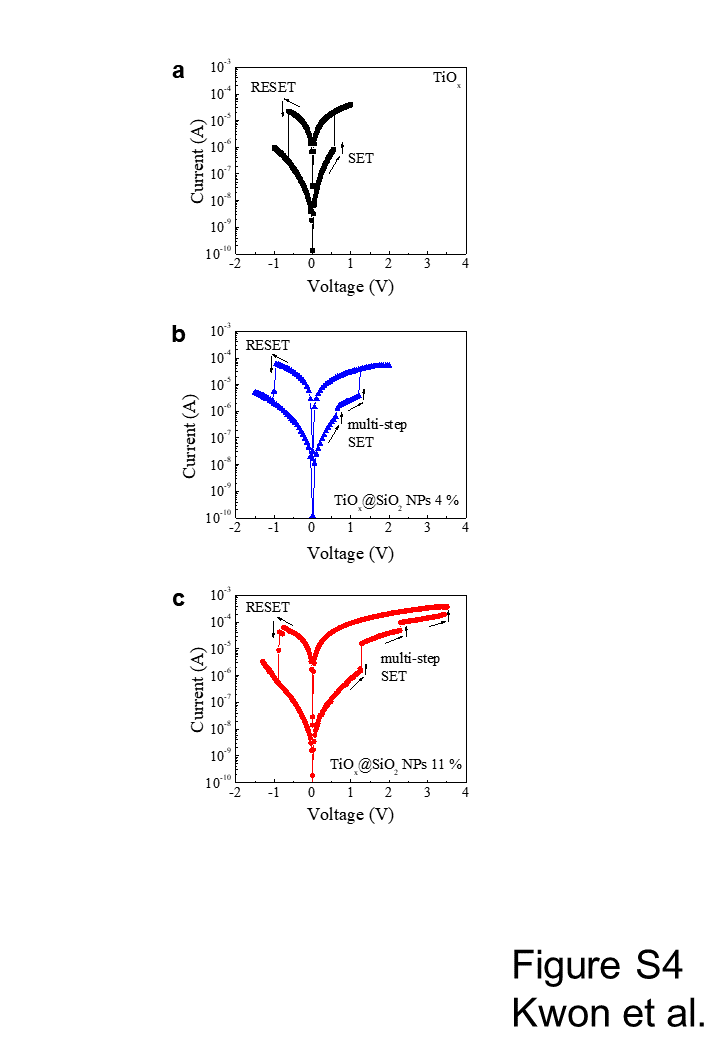


**Figure S4**. The resistive switching characteristics as a function of the composition of SiO_2_ NPs with (a) 0 %, (b) 4 %, and (c) 11 %.


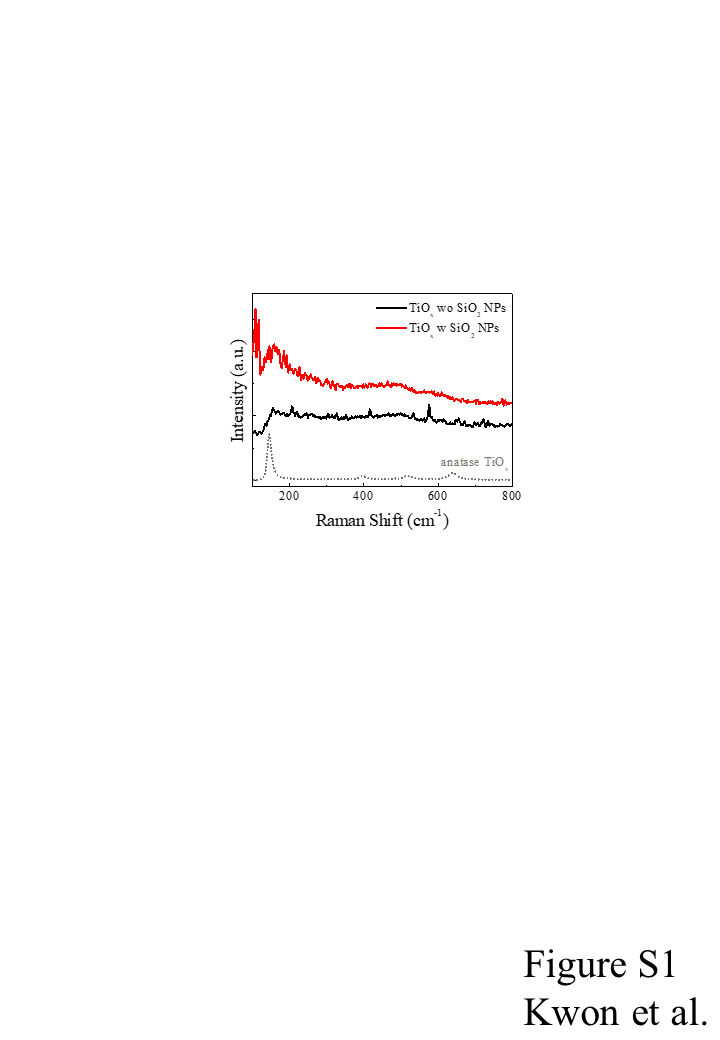


**Figure S5**. Raman spectra of TiO_x_ and TiO_x_@SiO_2_ NPs films. Dotted lines indicate anatase TiO_x_.
